# Icaritin attenuates 6-OHDA-induced MN9D cell damage by inhibiting oxidative stress

**DOI:** 10.7717/peerj.13256

**Published:** 2022-04-12

**Authors:** Xinyu Zhou, Nanqu Huang, Xiaoyi Hou, Li Zhu, Yiman Xie, Zhisheng Ba, Yong Luo

**Affiliations:** 1 Department of Neurology, The First People’s Hospital of Zunyi & Third Affiliated Hospital of Zunyi Medical University, Zunyi, Ghuizhou, China; 2 National Drug Clinical Trial Institution, The First People’s Hospital of Zunyi & Third Affiliated Hospital of Zunyi Medical University, Zunyi, Guizhou, China; 3 School of Medicine and Technology, Zunyi Medical University, Zunyi, Guizhou, China

**Keywords:** Icartin, 6-Hydroxydopamine, Nuclear factor erythroid 2 related factor 2, Heme oxygenase 1, Superoxide dismutase, Oxidative stress

## Abstract

**Background:**

We assessed whether ICT can alleviate 6-OHDA-induced cell damage *via* inhibition of oxidative stress by evaluating the protective effect of icaritin (ICT) against 6-hydroxydopamine (6-OHDA)-induced MN9D cell damage and further determined the mechanism by which ICT reduces oxidative stress.

**Methods:**

MN9D cells were treated with 6-OHDA, to study the mechanism underlying the neuroprotective effect of ICT. MN9D cell damage was assessed by the CCK-8 assay, flow cytometry was performed to measure the content of reactive oxygen species (ROS) in cells, a superoxide dismutase (SOD) kit was used to evaluate SOD activity, and Western blotting was used to measure the expression of α-synuclein (α-Syn), Tyrosine hydroxylase (TH), nuclear factor erythroid-2 related factor 2 (Nrf2), and heme oxygenase-1 (HO-1).

**Results:**

ICT reduced damage to MN9D cells induced by 6-OHDA. ICT increased SOD activity and TH expression and reduced ROS production and α-Syn expression. ICT promoted the translocation of Nrf2 from the cytoplasm to the nucleus and further increased the protein expression of HO-1.

**Conclusions:**

ICT protects against 6-OHDA-induced dopaminergic neuronal cell injury by attenuating oxidative stress, and the mechanism is related to modulate the activities of Nrf2, HO-1 protein, and SOD.

## Introduction

Parkinson’s disease (PD) is a progressive degenerative disease of the central nervous system (Labib et al., 2021) that mostly occurs in elderly individuals over the age of 65, with an incidence that increases with age (Ferrucci et al., 2020). The typical pathological change associated with PD is progressive degeneration of dopaminergic neurons in the substantia nigra pars compacta (SNPC) in the midbrain (Zheng et al., 2017). Current evidence suggests that oxidative stress is related to the pathogenesis of PD (Dionísio, Amaral & Rodrigues, 2021), and increased uperoxide dismutase (SOD), nuclear factor erythroid-2 related factor 2 (Nrf2) and heme oxygenase-1 (HO-1) signaling in the nervous system can reduce oxidative stress related to nerve damage (Nitti et al., 2018).

The brain is an organ extremely vulnerable to oxidative stress due to its high oxygen demand, high levels of polyunsaturated fatty acids that are vulnerable to free radical attack, and low levels of antioxidant enzymes. In familial and sporadic PD, oxidative stress leads to α-synuclein (α-Syn) misfolding, modification and aggregation, and lipid peroxidation. Nrf2 is the main regulator of cellular redox homeostasis and can regulate the redox state of the cell. It upregulates the expression of downstream antioxidant enzymes to resist oxidative stress and plays a vital role in the resistance of cells to reactive oxygen species (ROS) (Chen et al., 2017; Sun et al., 2021). Under physiological conditions, Nrf2 binds to Keap1 and exists in the cytoplasm, and Keap1 mediates the ubiquitination degradation of Nrf2 by the proteasome, thereby maintaining a physiological state of low Nrf2 activity (Kim et al., 2013; Loboda et al., 2016). When subjected to oxidative stress or other chemical stimuli, phosphorylated Nrf2 and Keap1 dissociate into the nucleus, form a heterodimer with the Maf protein, and then bind to the antioxidant response element (ARE), regulating the activity of their target genes such as SOD and HO-1 and thereby scavenging harmful molecules such as ROS and protecting cells from free radical damage (Huang et al., 2016; Villavicencio Tejo & Quintanilla, 2021). Activation of the expression of Nrf2 and HO-1 proteins can effectively reduce oxidative stress and prevent neuronal degeneration, thereby preventing the onset and progression of neurodegenerative diseases.

Epimedium is a plant belonging to the genus *Epimedium* of the Berberis family. It is a flavonoid compound that is an important natural antioxidant that can increase the levels of metabolic free radical enzymes (Angeloni, Barbalace & Hrelia, 2019; Liu et al., 2019). Icaritin (ICT) and icariin (ICA) are both effective ingredients extracted from epimedium. Studies have shown that ICA has a very prominent antioxidative stress effect in dopaminergic neurons. ICA can activate Nrf2 and respond positively to oxidative stress caused by ROS (Zhu et al., 2019). ICT is formed by cellulase-mediated hydrolyzation of ICA. The molecular formula of ICT is C_21_H_20_O_6_, and its molecular weight is 368.38. ICT has a lower molecular weight than ICA and more easily passes through the blood–brain barrier. ICT contains a polyphenolic hydroxyl group and can scavenge free radicals, thus delaying aging and exerting neuroprotection (Lai et al., 2013; Li et al., 2012; Sheng et al., 2013). However, there is currently little research on the detailed mechanism by which ICT protects dopaminergic neurons through antioxidant activity; thus, we conducted research on the underlying mechanism.

## Materials and Methods

### Reagents

ICT (HPLC analytical purity 99.5%) was purchased from Beijing Solarbio Technology Co., Ltd. (Beijing, China), and 6-hydroxydopamine (6-OHDA) was purchased from Sigma Chemical Co. (St. Louis, MO, USA). A CCK-8 assay kit was purchased from Apexbio (Apexbio, Houston, TX, USA), and total SOD and ROS detection kits were purchased from Beijing Soleibao Technology Co., Ltd. (Beijing, China). Anti-tyrosine hydroxylase (TH) (Ab112), anti-α-synuclein (α-Syn) (Ab52168), anti-Nrf2 (Ab156883) and anti-HO-1 (Ab13243) antibodies were purchased from Abcam (Cambridge, MA, USA), and anti-β-actin (20536-1-AP), anti-PCNA (10205-1-AP) and anti-GAPDH (10494-1-AP) antibodies were purchased from Proteintech Group (Wuhan, China).

### Cell culture and treatment

MN9D dopaminergic cells were purchased from the Chinese Type Culture Collection of Wuhan University. MN9D cells were cultured in RPMI-1640 complete medium containing 10% fetal bovine serum and 1% penicillin–streptomycin at 37 °C in an incubator containing 5% CO_2_. ICT was prepared in methanol solution, diluted with medium to the appropriate concentration, and stored at −20 °C. MN9D cells were pretreated with ICT and vitamin C (VC, 200 µM) for 1 h, and 6-OHDA (50 μM) was added for 24 h. In this experiment, VC was used as the positive control group because of its antioxidant effect. The cells were collected, and the levels of related indicators were assessed.

### Cell viability

MN9D cells were pretreated with ICT for 1 h, and 6-OHDA (50 μM) was added for 24 h. After 10 μl/well CCK-8 solution was added, the cells were incubated at 37 °C for 1 h, and then the absorbance was measured at 450 nm with a Synergy HTX microplate reader (Bio Tek, Winooski, VT, USA).

### ROS assay

Intracellular ROS levels were measured with the fluorescent probe dichlorodihydrofluorescein diacetate (DCFH-DA; Calbiochem, CA, USA). The MN9D cells were processed, incubated with DCFH-DA (2 μM) in a 37 °C incubator for 20 min, and collected, and intracellular ROS levels in all cells were measured by flow cytometry (488 nm for excitation, 525 nm for emission).

### SOD activity assay

MN9D cells were collected and centrifuged at 4 °C. After addition of reagents and incubated in a 37 °C Calorstat thermotank for 30 min, SOD levels were measured using the WST-8 method. Then, SOD activity was calculated according to the absorbance measured with a microplate reader at 450 nm.

### Western blot analysis

MN9D cells were lysed and centrifuged, and then equal amounts of protein were loaded on 10% Bis-Tris Nu-PAGE gels and transferred onto polyvinylidene difluoride membranes, which were blocked with 5% skimmed milk and incubated overnight at 4 °C with the following primary antibodies: Nrf2 (1 μg/ml), HO-1 (1:2,000), TH (1:200), α-Syn (1:1,000), GAPDH (1:5,000), PCNA (1:2,000), and β-actin (1:1,000). Finally, ECL reagents were used to visualize the membrane.

### Molecular docking

The protein crystal structure of Nrf2 (PDB ID: 2lz1) used for virtual docking was downloaded from the PDB database (https://www.rcsb.org). The 3D structure of ICT was downloaded from the PUBCHEM database. Energy minimization was then performed under the MMFF94 force field using Chem3D v20. In this study, AutoDock Vina 1.1.2 software was used for molecular docking work.

### Statistical analysis

The data are expressed as the mean ± SD. All data related to the experiment were analyzed by one-way analysis of variance (ANOVA) in SPSS 22.0 (IBM, Armonk, NY, USA). When ANOVA showed significant differences, pairwise comparisons between means were assessed by Bonferroni’s *post hoc* t test with correction. A value of *P* < 0.05 was considered statistically significant.

## Results

### ICT protects MN9D cells from 6-OHDA-induced cytotoxicity

**Figure 1:**
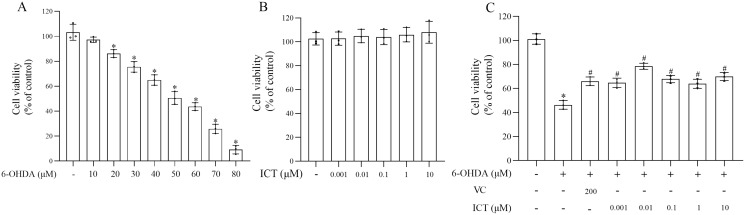
ICT protects neuronal cells against 6-OHDA-induced cytotoxicity. (A) Cytotoxicity in MN9D cells incubated with 10, 20, 30, 40, 50, 60, 70, or 80 µM 6-OHDA, as determined by the CCK-8 assay. (B) Cytotoxicity in MN9D cells incubated with 0.001, 0.01, 0.1, 1, or 10 µM ICT, as determined by the CCK-8 assay. (C) Cytotoxicity in MN9D cells treated with 0.001, 0.01, 0.1, 1, or 10 µM ICT, vitamin C (200 µM) and 50 µM 6-OHDA, as determined by the CCK-8 assay. The data are shown as the mean ± SD; *n* = 3 (*P < 0.05 *vs*. the control group, ^#^*P* < 0.05 *vs*. the 6-OHDA group).

MN9D cells were treated with 10, 20, 30, 40, 50, 60, 70, 80 µM 6-OHDA and 0.001, 0.01, 0.1, 1, 10 µM ICT to evaluate the cytotoxic effects of 6-OHDA and ICT. The CCK-8 assay showed that 6-OHDA (50 µM) reduced the viability of MN9D cells to 50.5% (Fig. 1A). ICT alone (0.001, 0.01, 0.1, 1, or 10 µM) had no cytotoxic effects (Fig. 1B). In addition, ICT (0.01 µM) and VC (200 µM) significantly reduced 6-OHDA (50 µM)-induced MN9D neuronal damage (Fig. 1C). These results indicate that ICT significantly inhibits 6-OHDA-induced neurotoxicity.

### ICT treatment can significantly reduce 6-OHDA-induced oxidative stress in MN9D cells

**Figure 2:**
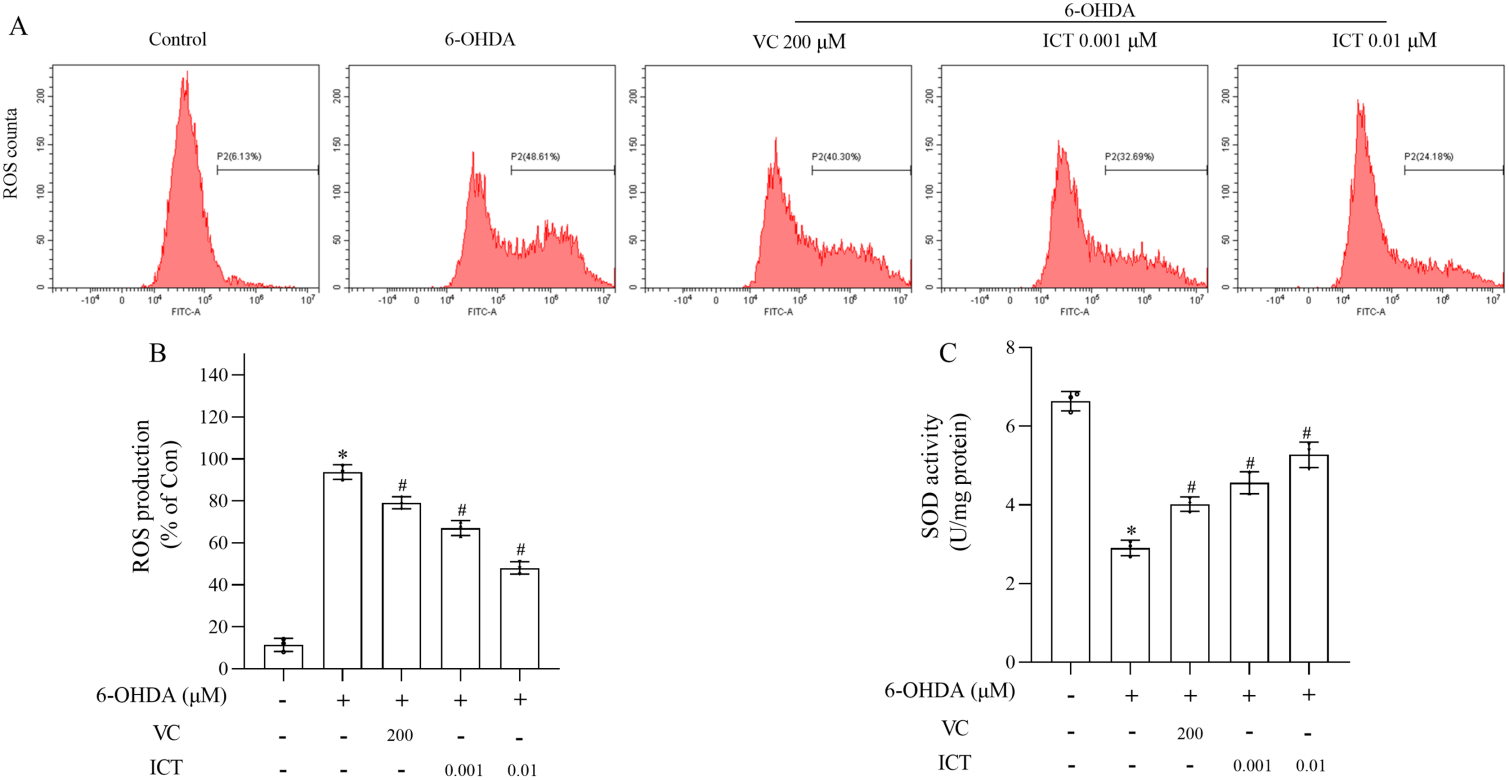
ICT significantly reduced oxidative stress induced by 6-OHDA in MN9D cells. (A) ROS levels. (B) ROS production. (C) SOD activity. The data are shown as the mean ± SD; *n* = 3 (*P < 0.05 *vs*. the control group, ^#^*P* < 0.05 *vs*. the 6-OHDA group).

The effect of ICT on 6-OHDA-induced cytotoxicity was investigated by measuring ROS levels and SOD activity. The level of ROS in MN9D cells treated with 6-OHDA was significantly increased, and the activity of SOD was significantly reduced. Compared with VC (200 µM), ICT (0.01 µM) significantly reduced the level of ROS in MN9D cell, and increased the activity of SOD (Figs. 2A–2C).

### ICT reduces the protein expression of α-Syn

**Figure 3:**
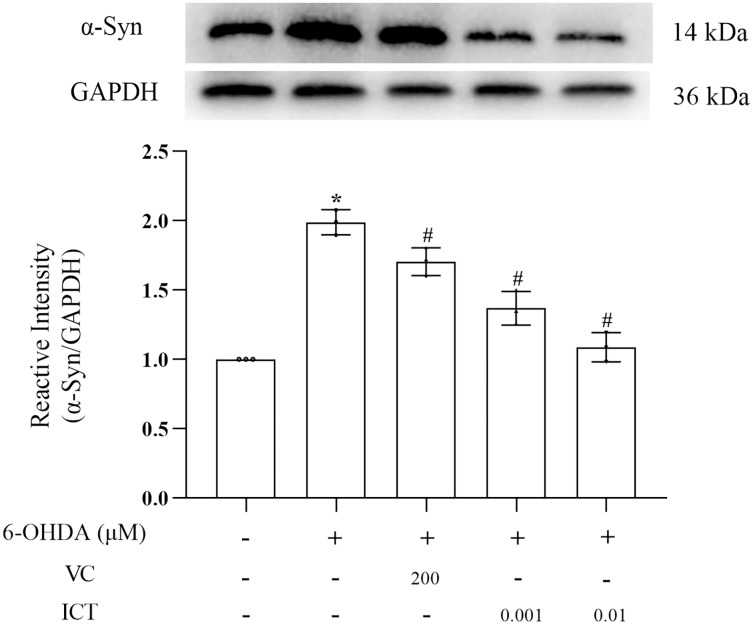
ICT reduces the protein expression of α-Syn. Western blotting was used to assess the protein expression level of α-Syn in MN9D cells treated with 0.001 and 0.01 µM ICT. The data are shown as the mean ± SD; *n* = 3 (*P < 0.05 *vs*. the control group, ^#^*P* < 0.05 *vs*. the 6-OHDA group).

α-Syn is a core focus of research on PD neuropathology. It is easily misfolded and aggregates into neurotoxic oligomers (Agliardi et al., 2021). The results showed that the level of α-Syn in MN9D cells treated with 6-OHDA was significantly increased. Compared with VC (200 µM), ICT (0.01 µM) significantly reduced the level of α-Syn in MN9D cells (Fig. 3).

### ICT increases the protein expression of TH

**Figure 4:**
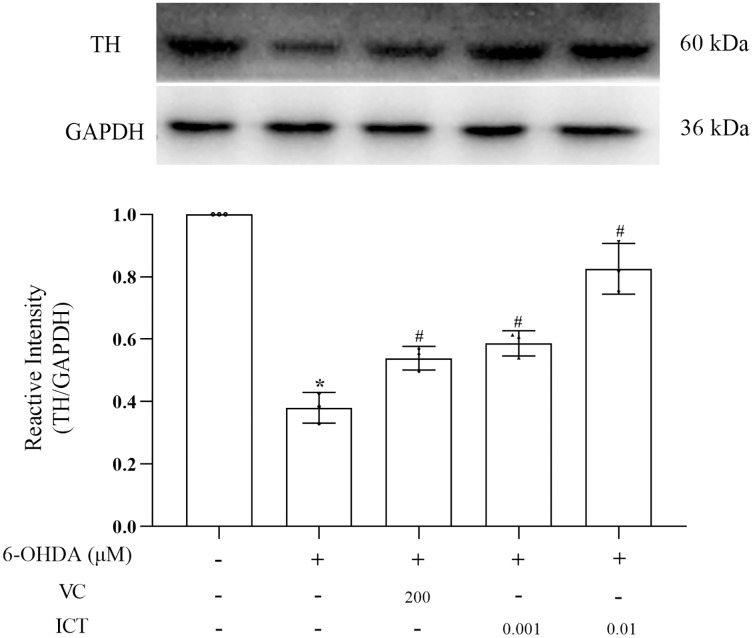
ICT increases the protein expression of TH. Western blotting was used to measure the TH protein expression level in MN9D cells treated with 0.001 and 0.01 µM ICT. The data are shown as the mean ± SD; *n* = 3 (*P < 0.05 *vs*. the control group, ^#^*P* < 0.05 *vs*. the 6-OHDA group).

The results showed that the level of TH in MN9D cells treated with 6-OHDA was significantly reduced. Compared with VC (200 µM), ICT (0.01 µM) significantly increased the level of TH in MN9D cells (Fig. 4).

### ICT exerts a protective effect by increasing the expression of Nrf2

**Figure 5:**
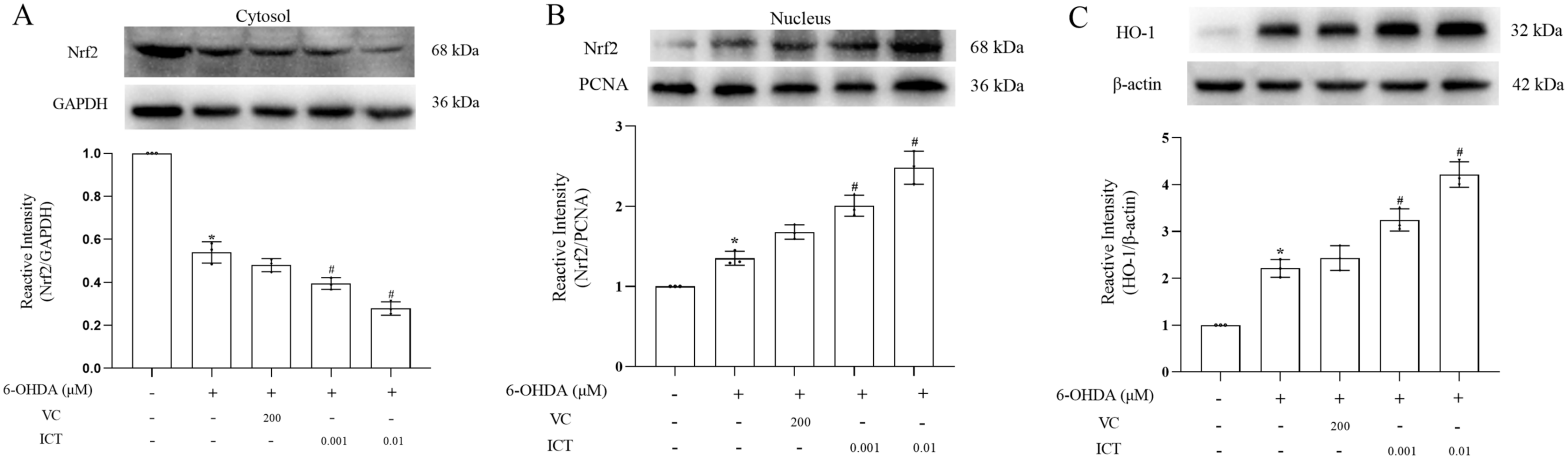
ICT exerts its protective effect by altering the expression of Nrf2/HO-1. (A) Nrf2/GAPDH expression. (B) Nrf2/PCNA expression. (C) HO-1 expression. The data are shown as the mean ± SD; *n* = 3 (*P < 0.05 *vs*. the control group, ^#^*P* < 0.05 *vs*. the 6-OHDA group).

We observed the effect of ICT on the protein expression of Nrf2. The results showed first that ICT (0.01 µM) increased the translocation of Nrf2 to the nucleus and significantly reduced the level of Nrf2 in the cytoplasm (Fig. 5A). Second, the expression of HO-1 was evaluated to determine whether ICT regulates the expression of genes downstream of Nrf2. As shown in Fig. 5B, after ICT (0.01 µM) treatment, the expression of HO-1 was upregulated, and the effect of ICT treatment was more significant than that of VC treatment. These data indicate that ICT can alleviate 6-OHDA-induced oxidative stress by modulating the Nrf2 pathway.

### ICT bound Nrf2

**Figure 6:**
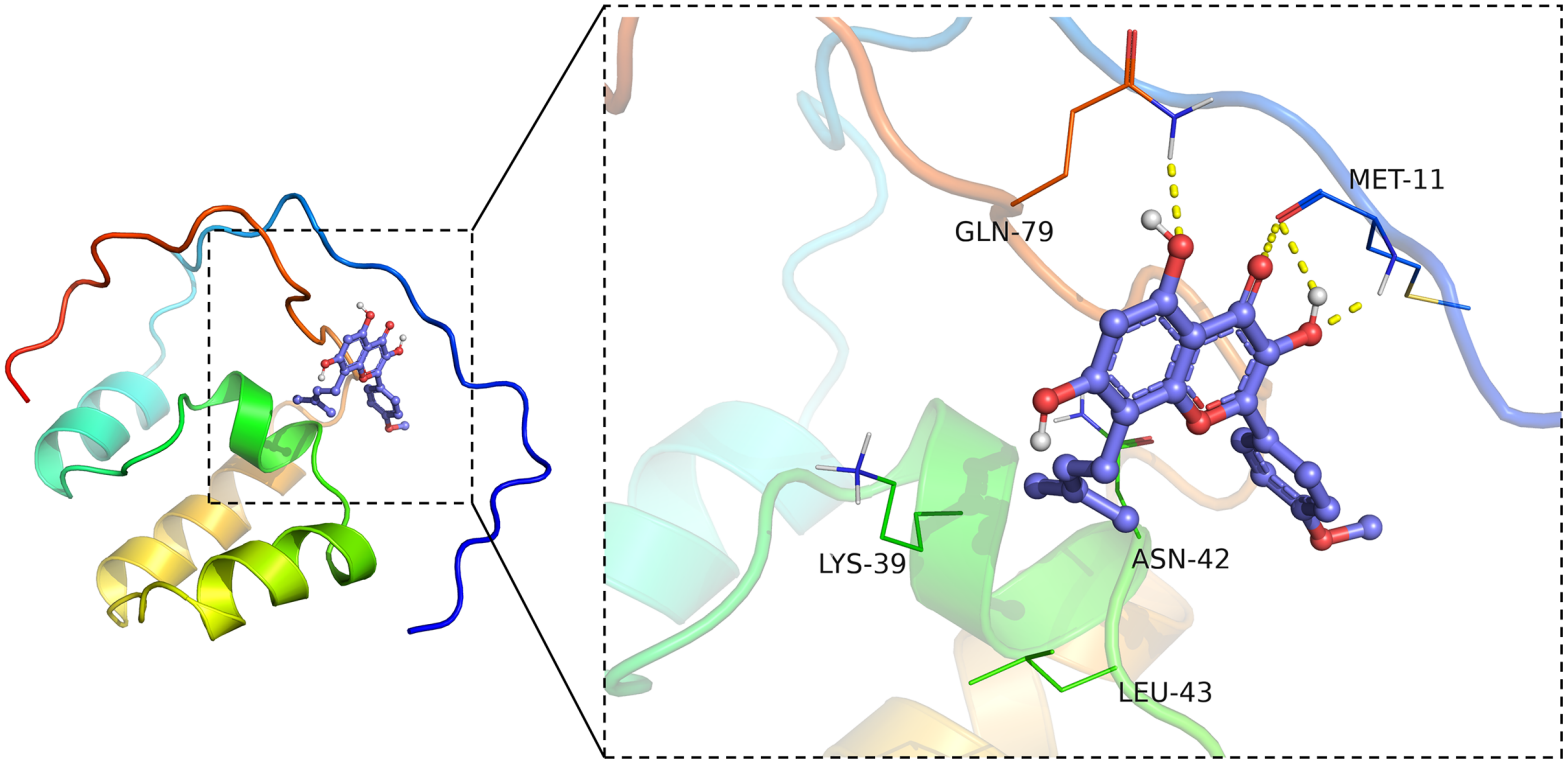
ICT binds Nrf2. The ICT-Nrf2 binding mode obtained based on virtual molecular docking. The green lines represent the amino acids around the binding site. The yellow dotted lines represent the hydrogen bond interaction.

Virtual molecular docking was adapted to verify whether ICT could bind Nrf2 protein. The results showed that crystal structure of the catalytic domain of Nrf2, a primary functional structure of Nrf2 protein, well combined with ICT according to their binding energy (−6.4 kcal/mol). Notably, the results also confirmed that ICT can form a total of four hydrogen bonds with GLN79 and MET11 main chain amide groups on Nrf2 protein, and hydrogen bonds may be the key interaction between Icaritin and Nrf2. The hydrocarbyl arm of ICT also forms hydrophobic interactions with ASN42, LEU43, and LYS39, which further strengthens the binding (Fig. 6). Taken together, the findings indicated that ICT might directly bind with Nrf2, thereby exerted neuroprotective effect.

## Discussion

In this study, we analyzed the effect of ICT in PD cell models. Previous experiments have shown that VC can upregulate the expression of Nrf2, HO-1; thus, VC was used in this experiment as a positive control group for studying the mechanism underlying the effect of ICT (Mostafavi-Pour et al., 2017; Wang et al., 2021; Wang et al., 2019). The results show that ICT can reduce ROS generation by regulating the expression of genes related to endogenous antioxidant systems (Nrf2 and HO-1) and the activity of SOD in MN9D cells, and the effect of ICT treatment is more than that of vitamin C treatment. Significantly. Through this mechanism, ICT reduces 6-OHDA-induced oxidative stress damage in dopaminergic neurons. This provides new insight into the neuroprotective mechanisms of ICT.

Previous experiments have shown that ICT can cross the blood–brain barrier, and numerous studies have shown that ICT also has a protective effect on cells in the central nervous system, suggesting that ICT plays an important role in the treatment of neurodegenerative diseases. ROS are generated during the maintenance of metabolic homeostasis in the body, as various antioxidant mechanisms stabilize the redox balance of cells, and steady-state changes can lead to excessive ROS production. Excessive ROS production leads to DNA mutations and protein denaturation and ultimately leads to damage to dopaminergic neurons (Hwang, 2013; Zuo & Motherwell, 2013). In this study, we used MN9D cells, which have the characteristics of dopaminergic neurons and express TH. It was also observed that ICT can reduce the level of ROS and increase the activity of the antioxidant enzyme SOD, significantly reducing oxidative stress damage to MN9D cells.

Furthermore, we observed that ICT can reduce the expression of α-Syn. Hashimoto M and Owen Scudamore found that oxidative stress can exacerbate the aggregation of α-Syn into neurotoxic oligomers *in vivo*, leading to the death of dopaminergic neurons (Scudamore & Ciossek, 2018). ICT can reduce oxidative stress in cells, thereby reducing the aggregation and production of α-Syn and reducing its toxic effect on dopaminergic neurons.

Studies have found that in an MPP^+^-induced PD cell model, the expression of TH in dopaminergic neurons is reduced (Höglinger et al., 2003; Wen et al., 2021). Therefore, we also observed the effect of ICT on TH expression. TH is a monooxygenase and the rate-limiting enzyme of the first step in the synthesis of levo-dopamine (L-DA), which catalyzes the formation of levodopa from levo-tyrosine and the removal of carboxyl groups to produce L-DA *via* catalysis by aromatic decarboxylases. Therefore, TH is very important in the synthesis of dopamine, and a lack or insufficient expression of TH directly affects the synthesis and secretion of dopamine (Mirzaeipour et al., 2021; Ranjbar et al., 2021). In this experiment, we found that ICT increases TH expression and may further increase dopamine synthesis, in turn exerting protective effects on neurons.

Based on the antioxidative stress effect of ICT, we also studied the changes of Nrf2 and HO-1 proteins in MN9D cells treated with ICT. Nrf2 and HO-1 proteins are key regulatory pathways of oxidative stress (Kim, Cha & Surh, 2010). When oxidative stress occurs, Keap1 is modified and Nrf2 is released from Keap1 so that it is not degraded by ubiquitination, its expression remains stable, and it remains inactive. Meanwhile, Nrf2 is phosphorylated, aggregates in the nucleus, dimerizes with Maf proteins, and then activates the transcription of its target genes upon binding to ARE sequences, regulating the expression of antioxidant genes such as HO-1 expression, reducing ROS production and increasing the activity of the antioxidant enzyme SOD (Ben-Yehuda Greenwald et al., 2017; Wakabayashi et al., 2010). As expected, ICT increased Nrf2 translocation into the nucleus while decreasing the level of Nrf2 in the cytoplasm, indicating that ICT promotes Nrf2 nuclear translocation and increases the expression of HO-1, thereby attenuating 6-OHDA-induced oxidative stress.

This study proved that ICT can counteract the neurotoxicity induced by 6-OHDA. Our findings suggest that ICT may modulate the activities of Nrf2, HO-1 protein, and SOD, activate endogenous protective mechanisms against oxidative stress injury, and thus provide viable neuroprotectants and potential treatments for neurodegenerative diseases.

## Conclusions

In conclusion, this study shows that ICT regulate the expression of Nrf2, HO-1 and increases the activity of the antioxidant enzyme SOD, eliminating excessive ROS. Therefore, ICT can protect dopaminergic neurons by reducing oxidative stress caused by 6-OHDA. This study provides further evidence that ICT may be a potential drug for the treatment of neurodegenerative diseases.

## Supplemental Information

10.7717/peerj.13256/supp-1 Supplemental Information 1 Raw data for Figs. 1–5. All results are expressed as the mean ± SD. The difference between the means of more than two groups was analyzed by one-way analysis of variance (ANOVA). When ANOVA showed significant differences, pairwise comparisons of means were made by Bonferroni’s post hoct-test with correction. A value of *P* < 0.05 was considered statistically significant.

10.7717/peerj.13256/supp-2 Supplemental Information 2 Full-length uncropped blots of Figs. 3–5. The MN9D cells were lysed and centrifuged, and the protein in the supernatant was quantified using the BCA kit. Add an equal amount of total protein to 10% Bis-Tris Nu-PAGE Gel, then transfer to a polyvinylidene fluoride membrane, seal the membrane with 5% skim milk, and incubate with the following primary antibodies at 4 °C Overnight: Anti-Nrf2 (1 μg/ml), HO-1 (1:2,000), TH (1:200), α-Syn (1:1,000), GAPDH (1:5,000), PCNA (1:2,000), β-actin(1:1,000), wash the membrane 3 times with TBST, Finally, High-Sig ECL Western Blotting Substrate (Shanghai Tanon Technology Co., Ltd., Shanghai, China) was used to visualize the membrane.

## Additional Information and Declarations

### Data Availability

The following information was supplied regarding data availability: The raw measurements are available in the Supplemental Files.
