# Supplementary material for: Icaritin attenuates 6-OHDA-induced MN9D cell damage by inhibiting oxidative stress
Source: PeerJ. 2022 Apr 12;10:e13256. doi: 10.7717/peerj.13256 (PMC9012182; doi:10.7717/peerj.13256)
Supplement: Supplemental Information 2 — The MN9D cells were lysed and centrifuged, and the protein in the supernatant was quantified using the BCA kit. Add an equal amount of total protein to 10% Bis-Tris Nu-PAGE Gel, then transfer to a polyvinylidene fluoride membrane, seal the membrane with 5% skim milk, and incubate with the following primary antibodies at 4 °C Overnight: Anti-Nrf2 (1 μg/ml), HO-1 (1:2,000), TH (1:200), α-Syn (1:1,000), GAPDH (1:5,000), PCNA (1:2,000), β-actin(1:1,000), wash the membrane 3 times with TBST, Finally, High-Sig ECL Western Blotting Substrate (Shanghai Tanon Technology Co., Ltd., Shanghai, China) was used to visualize the membrane. [file peerj-10-13256-s002.docx]

**Full-length uncropped blots**

**Materials and methods of Western blot assay**

The MN9D cells were lysed and centrifuged, and the protein in the supernatant was quantified using the BCA kit. Add an equal amount of total protein to 10% Bis-Tris Nu-PAGE Gel, then transfer to a polyvinylidene fluoride membrane, seal the membrane with 5% skim milk, and incubate with the following primary antibodies at 4°C Overnight: Anti-Nrf2 (1μg/ml), HO-1 (1:2,000), TH (1:200), α-Syn (1:1,000), GAPDH (1:5,000), PCNA (1:2,000), β-actin(1:1,000), wash the membrane 3 times with TBST, Finally, High-Sig ECL Western Blotting Substrate (Shanghai Tanon Technology Co., Ltd.) was used to visualize the membrane.

Figure 3. ICT mediates its protective effect by reducing the expression of α-Syn protein by reducing oxidative stress.


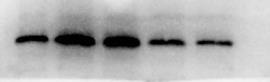


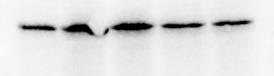


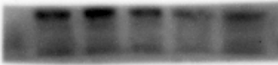


α-Syn-From left to right are Control, 6-OHDA, VC+6-OHDA, 0.001µM ICT+6-OHD, 0.01µM ICT+6-OHD group.


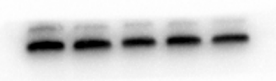


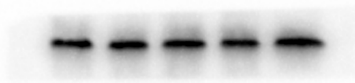


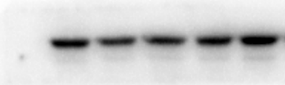


GAPDH-From left to right are Control, 6-OHDA, VC+6-OHDA, 0.001µM ICT+6-OHD, 0.01µM ICT+6-OHD group.

Figure 4. ICT mediates its protective effect by reducing the expression of TH protein by reducing oxidative stress.


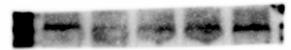


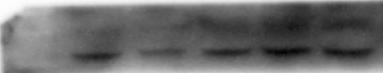


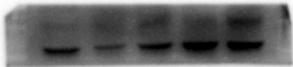


TH-From left to right are Control, 6-OHDA, VC+6-OHDA, 0.001µM ICT+6-OHD, 0.01µM ICT+6-OHD group.


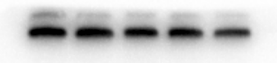


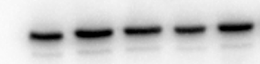


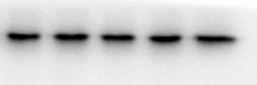


GAPDH-From left to right are Control, 6-OHDA, VC+6-OHDA, 0.001µM ICT+6-OHD, 0.01µM ICT+6-OHD group.

Figure 5. ICT mediates its protective effect by changing the expression of Nrf2 related antioxidant genes.


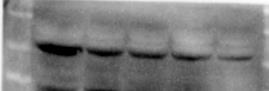


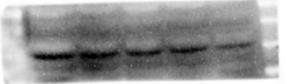


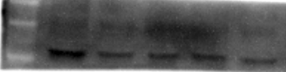


Nrf2 Cytosol-From left to right are Control, 6-OHDA, VC+6-OHDA, 0.001µM ICT+6-OHD, 0.01µM ICT+6-OHD group.


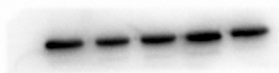


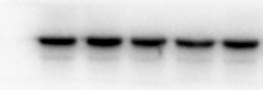


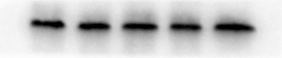


GAPDH-From left to right are Control, 6-OHDA, VC+6-OHDA, 0.001µM ICT+6-OHD, 0.01µM ICT+6-OHD group.


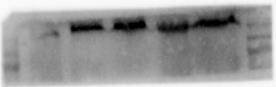


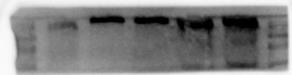


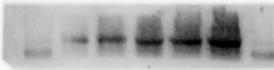


Nrf2 Nucleus-From left to right are Control, 6-OHDA, VC+6-OHDA, 0.001µM ICT+6-OHD, 0.01µM ICT+6-OHD group.


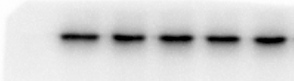


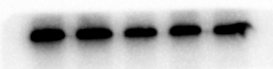


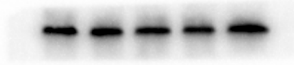


PNCA-From left to right are Control, 6-OHDA, VC+6-OHDA, 0.001µM ICT+6-OHD, 0.01µM ICT+6-OHD group.


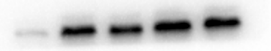


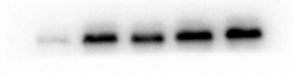


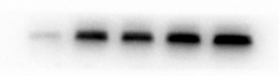


HO-1-From left to right are Control, 6-OHDA, VC+6-OHDA, 0.001µM ICT+6-OHD, 0.01µM ICT+6-OHD group.


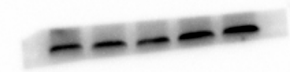


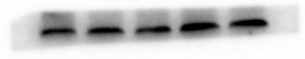


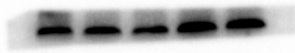


β-actin-From left to right are Control, 6-OHDA, VC+6-OHDA, 0.001µM ICT+6-OHD, 0.01µM ICT+6-OHD group.
